# Supplementary material for: The causal role of circulating inflammatory markers in osteoporosis: a bidirectional Mendelian randomized study
Source: Front Immunol. 2024 Jul 18;15:1412298. doi: 10.3389/fimmu.2024.1412298 (PMC11291241; doi:10.3389/fimmu.2024.1412298)
Supplement: Supplementary file 1 [file DataSheet_1.zip › supplementary material/OSTEOPOROSIS-CIM/GCST90274802.tsv.gz_leave_one_out.pdf]

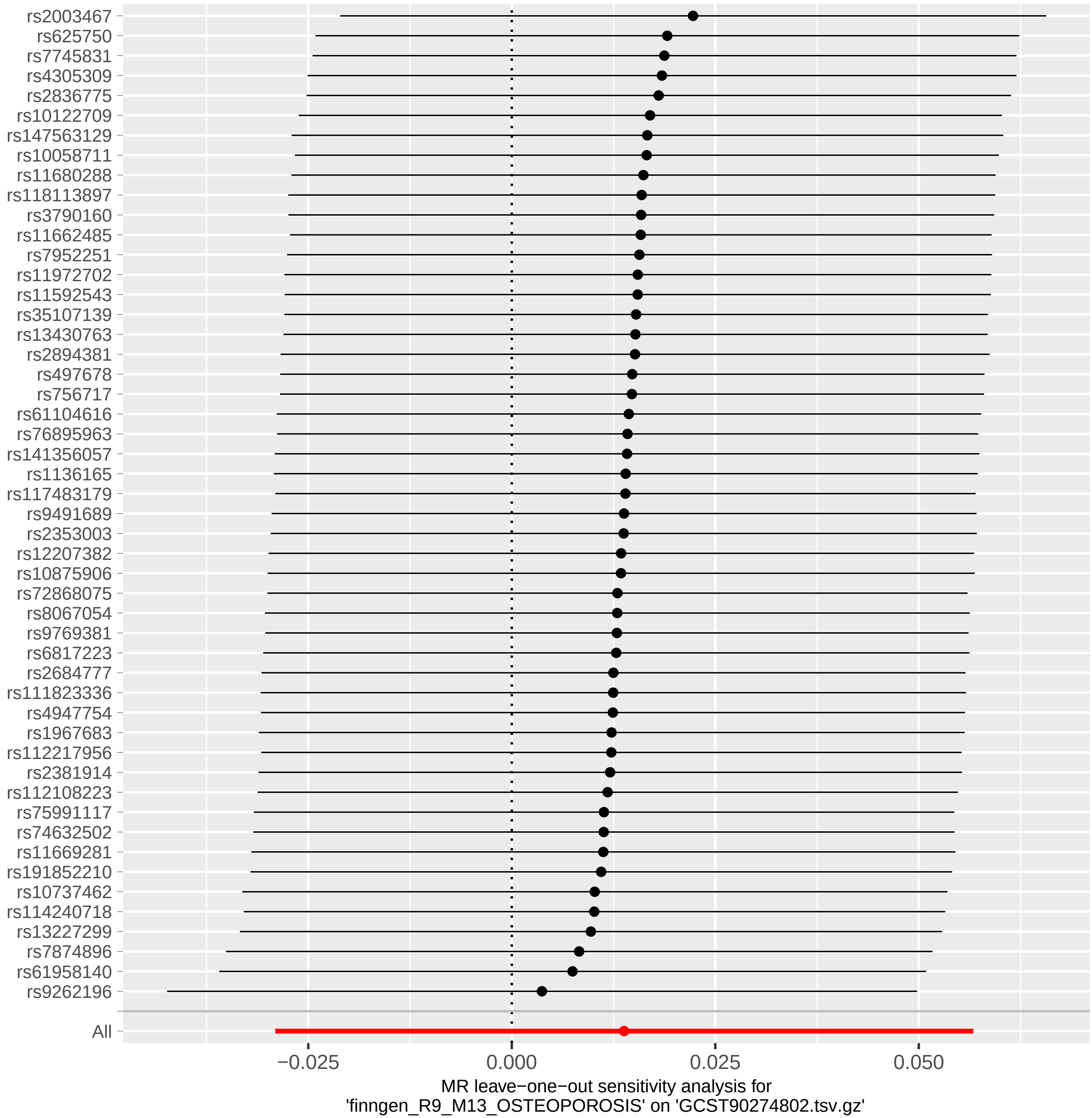
rs2003467
rs625750
rs7745831
rs4305309
rs2836775
rs10122709
rs147563129
rs10058711
rs11680288
rs118113897
rs3790160
rs11662485
rs7952251
rs11972702
rs11592543
rs35107139
rs13430763
rs2894381
rs497678
rs756717
rs61104616
rs76895963
rs141356057
rs1136165
rs117483179
rs9491689
rs2353003
rs12207382
rs10875906
rs72868075
rs8067054
rs9769381
rs6817223
rs2684777
rs111823336
rs4947754
rs1967683
rs112217956
rs2381914
rs112108223
rs75991117
rs74632502
rs11669281
rs191852210
rs10737462
rs114240718
rs13227299
rs7874896
rs61958140
rs9262196
All
−0.025
0.000
0.025
0.050
MR leave−one−out sensitivity analysis for
'finngen_R9_M13_OSTEOPOROSIS' on 'GCST90274802.tsv.gz'
